# An Uncommon Case of Multiple, Recurrent Cerebrospinal Fluid Venous Fistulas

**DOI:** 10.7759/cureus.49496

**Published:** 2023-11-27

**Authors:** May Ameri, Andrew Whyte, Stephen Chen, Andrew G Lee, Nagham Al Zubidi

**Affiliations:** 1 Department of Ophthalmology, University of Texas Health Science Center at Houston, Houston, USA; 2 Department of Head and Neck Surgery, University of Texas MD Anderson Cancer Center, Houston, USA; 3 Department of Interventional Radiology, University of Texas MD Anderson Cancer Center, Houston, USA; 4 Department of Ophthalmology, Houston Methodist Blanton Eye Institute, Houston, USA; 5 Department of Investigational Cancer Therapeutics, Division of Cancer Medicine, University of Texas MD Anderson Cancer Center, Houston, USA

**Keywords:** headache disorders, diplopia, spontaneous intracranial hypotension, ct myelogram, csf venous fistula

## Abstract

We present a clinical case to discuss the use of computed tomography (CT) spine myelograms alongside a complete history to diagnose multiple cerebrospinal fluid (CSF) venous fistulas (CVFs). The goal of this study is to familiarize clinicians with this challenging diagnosis and the utility of these studies in localizing leaks. A 63-year-old male patient with a history of cervical spinal stenosis, intermittent double vision, and sinus pressure managed with intermittent steroids presented to the clinic. He provided a detailed timeline of his previous symptoms and previous workups leading to the suspicion of intracranial hypotension due to CSF leak vs. CVF. Our workup, including magnetic resonance imaging (MRI) of the cervical spine and lumbar puncture (LP), was conducted. A CT thoracic spine myelogram was completed to localize the fistula site which was followed by the embolization of the fistula. The patient revealed complete resolution of his symptoms confirmed by imaging done one week postoperatively. This was a difficult case complicated by chronic misdiagnosis and confounding factors. CVFs were first described less than a decade ago; however, they are an extremely important cause of spontaneous intracranial hypotension. CVFs can be challenging to detect on conventional anatomical imaging like MRI. Thus, CT myelogram studies and a thorough history are crucial for accurate diagnosis. It is essential that clinicians, including ophthalmologists, learn to recognize CVFs as a potential cause of intracranial hypotension and become familiar with this diagnosis and its workup in the hopes that, unlike this case, the diagnosis and resolution of patients' life-altering symptoms are not delayed.

## Introduction

The first reported case of cerebrospinal fluid (CSF) venous fistulas (CVFs) in English literature was in 2014; however, they are now thought to be the cause of spontaneous intracranial hypotension (SIH) in 25% of patients [1]. Prior to 2014, known causes of SIH were dural tears and significant trauma, easily identifiable on magnetic resonance imaging (MRI) [1]. The suggested mechanism of CVF leading to SIH is the rupture of an arachnoid villi at the level of a nerve root sheath, leading to a direct connection between the CSF and a paravertebral venous system, allowing a unidirectional CSF flow from the CVF and lowering the pressure of the subarachnoid space [2]. Symptoms can include double vision, headaches, and tinnitus [3,4]. Most reported cases of CVFs occur in the thoracic spine; only two cases have been reported in the cervical spine [2]. Treatment of CVFs includes epidural blood patch, surgical ligation, embolization, fibrin glue injection, or a combination of any of the four [2]. We present a rare case of a patient with longstanding atypical, relapsing, and clinically significant SIH for over 20 years who was finally diagnosed with recurrent CVFs and successfully treated with surgical embolization. The goal of this study is to highlight the complexity of diagnosis, the use of dynamic lateral computed tomography (CT) myelograms, and the treatment of the manifestations of this relatively rare condition.

## Case presentation

**Figure 1:**
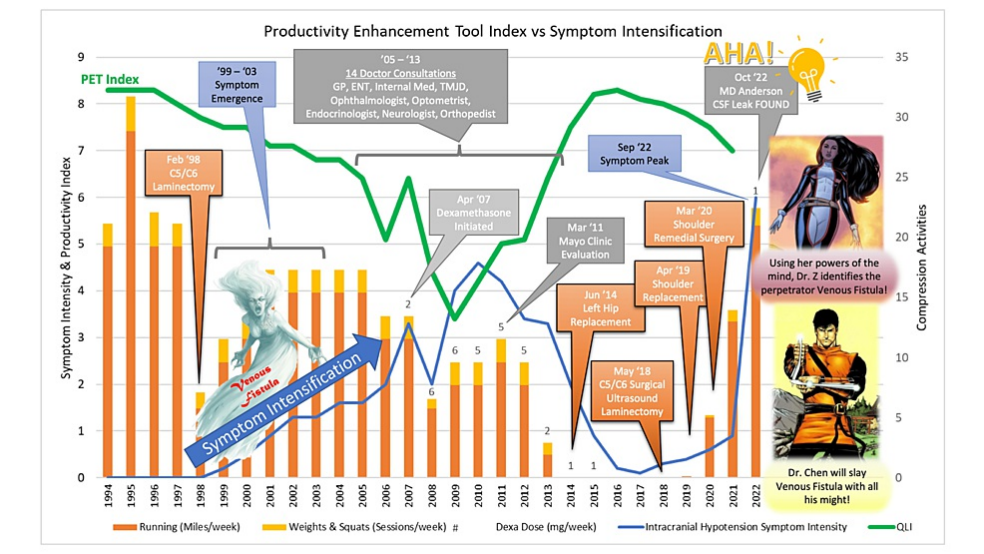
Patient perspective A patient-made timeline and detailed graph relaying patient's self-reported symptoms, diagnosis, and treatments

A 63-year-old male patient with a 20-year history of cervical (C4-C6) spinal stenosis and two previous laminectomies presents with chronic intermittent double vision, sinonasal pressure and pain, and tinnitus. He provided a detailed timeline of his previous symptoms and previous workups (Figure 1).

**Figure 2:**
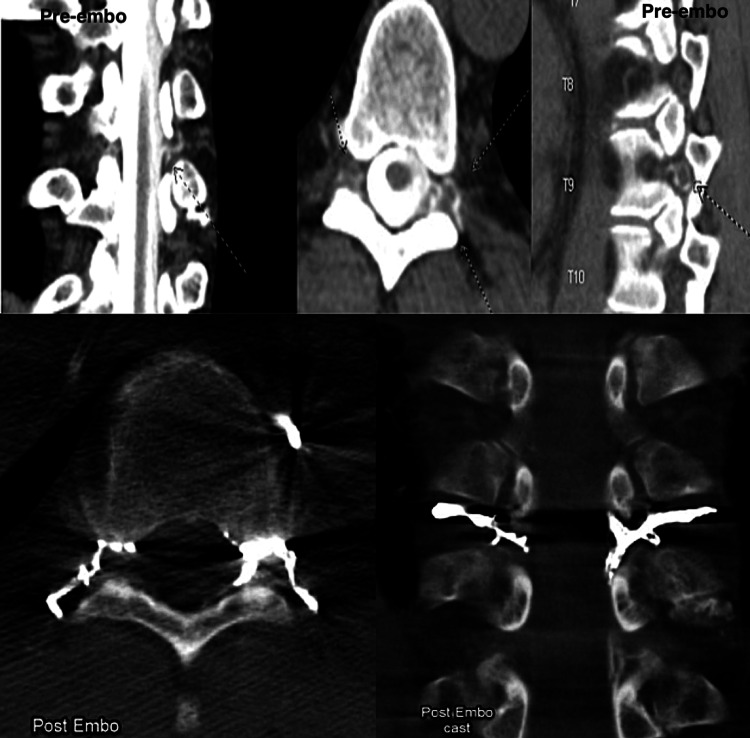
CT myelogram showing CVF suggestive of the source of CSF leak at T9-T10 CT: computed tomography; CVF: cerebrospinal fluid venous fistula; CSF: cerebrospinal fluid Top: Pre-embolization cast axial and sagittal images from CT myelography performed with the patient in lateral decubitus position showing drainage into the segmental spinal vein at T9, T10, and T11. Bottom: Post-embolization cast axial and sagittal images of CT myelography performed with the patient in lateral decubitus position showing venous embolization of T8, T10, and T11 spinal radicular veins

He reported subjective increased intensity of symptoms and progressive decrease in quality of life for 14 years until dexamethasone was started. Intermittent steroid use improved but did not resolve his symptoms, and he eventually became refractory to dexamethasone. Previous workup included magnetic resonance angiography (MRA), magnetic resonance venography (MRV) of the brain and spine, and lumbar puncture (LP); myelomalacia was detected alongside cord signal abnormality at C4-C6, but neither imaging nor LP showed specific signs of a cerebrospinal leak. Finally, a CT spine myelogram was conducted, which confirmed and localized two CVFs at T9-T10 and T11-T12 (Figure 2). A diagnosis of CVF was made, and transvenous embolization of T9-T10 and T11-T12 spinal radicular veins was performed. One-week post-op, the patient revealed complete resolution of his symptoms which was confirmed by post-op imaging (Figure 2). However, six months after embolization, the patient developed headache and diplopia with new CT myelogram findings confirming the recurrence of the CVF. The patient then underwent transcatheter venous embolization of T8, T10, and T11 spinal radicular veins and blood patch via LP at the level of T12-L1 with stabilization of his symptoms.

## Discussion

**Figure 3:**
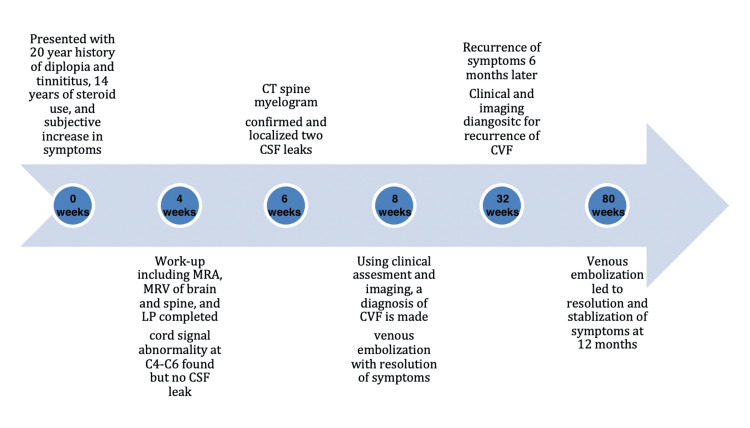
Timeline of symptoms, diagnostic assessment, and follow-up C: cervical spine; CSF: cerebrospinal fluid; MRA: magnetic resonance angiography; MRV: magnetic resonance venography; LP: lumbar puncture

Figure 3 shows the timeline of symptoms, diagnostic assessment, and follow-up.

This was an extremely difficult case complicated by chronic misdiagnosis and confounding factors. Strengths of this case include the detailed history provided by the patient and ability to track symptomology closely, while limitations are the possible confounding treatments of long-term steroid use, not typical for the treatment of CVF. A literature review found that 42% of intracranial hypotension patients presented with ocular manifestations. CVFs were only first ever documented in 2014; however, they are an extremely important cause of SIH [1]. Similar to other documented cases of CVFs including a case report by Hsieh et al. and a case series of 42 patients by Duvall et al., our patient presented with the common symptoms of headache, tinnitus, and double vision [3,4]. Specifically, in 77% of patients reported by Duvall et al., new-onset Valsalva-induced headache was the first and most common presenting symptom. Duvall et al. also found that in 40/42 patients, fistulas were located in the thoracic region between T7 and T12 and only 2/42 patients had CVFs located in the cervical spine, both of them located between C7 and T1 [4]. On the other hand, Hseih et al. reported a case of a 30-year-old woman with a history of Evans syndrome who presented with new-onset Valsalva-induced headache accompanied by mental fog, nausea, and a dragging sensation for four months [3]. She underwent a CT myelogram of the thoracic and cervical spine and was found with bilateral CVFs between C6 and C7 and was treated with surgical ligation. Thus, CVFs should be considered early in the differential diagnosis of new-onset Valsalva-induced (coughing, sneezing, laughing, straining) headaches or new-onset postural headaches in adults. Besides double vision, other reported ocular manifestations of SIH include visual acuity and field deficits, nystagmus, and photophobia [5]. Less commonly, cranial nerve palsies, most reported being abducens (VI) nerve palsy, and ophthalmoplegia have also been reported [5]. Further, CVFs can sometimes manifest with unique clinical presentations besides SIH, including frontotemporal brain sagging syndrome, subdural hygromas, and hemosiderosis [6,7]. Treatment options include epidural blood patch, surgical ligation, and fibrin glue; however, it has been shown that epidural blood patching is not as useful and often fails as a treatment for CVFs [8]. In comparison to other cases in English medical literature, our case is atypical due to the longstanding history of the patient's SIH presentation and the recurrence of multiple CVFs in both the cervical and thoracic spine. To our knowledge, this is only the third paper in English literature of a patient with multiple CVFs in the cervical spine and the first case of such a prolonged, atypical presentation [8,9].

## Conclusions

CVFs can be challenging to detect on conventional anatomic imaging because, unlike other types of spinal CSF leak, they do not typically result in the pooling of fluid in the epidural space. Thus, imaging studies like CT myelogram and thorough history, as seen in this patient, become vital. It is essential that ophthalmologists are familiarized with this diagnosis and its workup in the hopes that, unlike this case, the diagnosis and treatment of patients' life-altering symptoms are not delayed.
